# Research Progress of Antibody–Drug Conjugate Therapy for Advanced Gastric Cancer

**DOI:** 10.3389/fonc.2022.889017

**Published:** 2022-05-26

**Authors:** Na Wang, Qingyun Mei, Ziwei Wang, Lu Zhao, Dou Zhang, Dongying Liao, Jinhui Zuo, Hongxia Xie, Yingjie Jia, Fanming Kong

**Affiliations:** ^1^Department of Oncology, First Teaching Hospital of Tianjin University of Traditional Chinese Medicine, Tianjin, China; ^2^National Clinical Research Center for Chinese Medicine Acupuncture and Moxibustion, Tianjin, China

**Keywords:** gastric cancer, antibody–drug conjugates, targeted therapy, HER2, drug resistance

## Abstract

Gastric cancer is an intractable malignant tumor that has the fifth highest morbidity and the third highest mortality in the world. Even though various treatment options did much to ameliorate the prognosis of advanced gastric cancer, the survival time remained unsatisfactory. It is significant to develop new therapeutic agents to improve the long-term outcome. Antibody–drug conjugate is an innovative and potent antineoplastic drug composed of a specifically targeted monoclonal antibody, a chemical linker, and a small molecule cytotoxic payload. Powerful therapeutic efficacy and moderate toxicity are its preponderant advantages, which imply the inevitable pharmaceutical developments to meet the demand for individualized precision therapy. Nevertheless, it is unavoidable that there is a phenomenon of drug resistance in this agent. This article systematically reviewed the recent progress of antibody–drug conjugates in advanced gastric cancer therapy.

## Introduction

Gastric cancer (GC) is a common malignancy and remains a serious threat to human health worldwide. According to the 2021 statistics from the American Cancer Society, there were approximately 26,600 new GC cases and 11,200 deaths in the United States (1). Most GC patients were in the advanced stage when diagnosed and had a relatively poor prognosis (2). Compared with conventional medical treatments, antibody–drug conjugates (ADCs) have significant antitumorigenic ability and decreased systemic toxicity.

ADCs connect cytotoxic payloads to specific monoclonal antibodies (mAbs) through chemical linkers. The appropriate target antigens, which are supposed to be highly expressed in tumor cells, are most important in the optimization of ADCs (3). The humanized mAbs including IgG1, IgG2, and IgG4 subtypes are used to decrease the immunogenicity of ADCs. IgG1 is extensively used owing to the induction of antibody-dependent cell-mediated cytotoxicity (ADCC) (4). Stable linkers can maintain the integrity of ADCs, which ensures that ADCs do not release cytotoxic drugs early before reaching the target antigen to minimize off-target toxicity. Once ADCs are internalized, the linkers should have the ability to cleave quickly and release efficient cytotoxic drugs into the tumor cells (5). Cytotoxic drugs mainly consist of DNA damaging agents and tubulin inhibitors. The former generally combines with DNA double helix resulting in inhibition of DNA synthesis, DNA double-strand breaks, and apoptotic cell death. The latter commonly binds to microtubules to prevent microtubule polymerization, thereby blocking the cell cycle and inducing tumor cell apoptosis (6). ADCs bind to specific antigens on the surface of target cells after blood circulation, and the ADC-containing complex is internalized *via* clathrin-mediated endocytosis. Because of the different extent of organelle acidification, the complex is transported to the lysosomes or endosomes. The acid-cleavable linker is usually cracked in the early endosome and the protease-cleavable linker is eventually degraded by various proteases in the late endosome or lysosome (7, 8). The released cytotoxic agents play a vital role in killing tumor cells. After the Fab segment of mAb binds to the antigenic epitope of tumor cells, its Fc segment binds to Fc receptors on the surface of killer cells (e.g., natural killer cells, macrophages) to initiate ADCC. It mediates killer cells to kill tumor cells directly, inhibits the downstream signal transduction of antigen receptors, and releases pro-apoptotic proteins including perforin and granzyme to induce cancer cell death (9–11).

ADCs have demonstrated prospective landscapes in clinical research of advanced gastric cancer (AGC) (12). The present review mainly focused on the latest advances in ADCs targeting five specific targets including human epidermal growth factor receptor 2 (HER2), guanylyl cyclase C (GCC), Trop-2, etc. (**Table 1**).

**Table 1:** Selected ADCs in the review.

Name	Target	mAb	Linker	Payload	Efficacy
T-DM1 (13)	HER2	Trastuzumab	Non-cleavable MCC linker	DM1	mOS: 7.9 months; mORR: 20.6%
DS-8201a (14)	HER2	IgG1 mAb	Cleavable tetrapeptide linker	DXd	mORR: 51%; mOS: 12.5 months
RC48 (15)	HER2	Hertuzumab	Cleavable dipeptide linker	MMAE	mORR: 24.8%; mPFS: 4.1 months; mOS: 7.9 months
TAK-264 (16)	GCC	IgG1 mAb	Cleavable tetrapeptide linker	MMAE	NA
IMMU-132 (17)	Trop-2	RS7	Cleavable CL2A linker	SN-38	NA
LZ1904 (18)	CLDN18.2	LZ1903	Cleavable linker	9106-IM-2	–
EV20/NMS-P945 (19)	HER3	EV20	Cleavable linker	NMS-P528	–

mAb, monoclonal antibody; mOS, median overall survival; mORR, median objective response rate; mPFS, median progression-free survival; NA, not available.

## ADCs for the Treatment of AGC

### ADCs Targeting HER2

HER2 overexpression is accounting for approximately 15% to 20% of GC or gastroesophageal junction (GEJ) cancer (20). The heterogeneous expression of HER2 is common in GC and there are several HER2-targeted ADCs in clinical applications.

#### Trastuzumab Emtansine

Trastuzumab emtansine (T-DM1) is comprised of the mAb trastuzumab, a non-cleavable thioether linker, and the potent microtubule inhibitor emtansine (DM1) with a drug-to-antibody ratio (DAR) of 3.5 (21, 22). In preclinical studies, T-DM1 was found to be effective in killing trastuzumab-resistant cells and had low membrane permeability (23). T-DM1 has been approved by the Food and Drug Administration (FDA) for the therapy of HER2+ advanced breast cancer patients based on the data of the EMILIA and TH3RESA trials (24, 25).

The efficacy and safety of T-DM1 in HER2+ AGC patients were investigated by the GATSBY study (phase II/III, NCT01641939). The results showed that the median overall survival (mOS) [mOS: 7.9 vs. 8.6 months, HR: 1.15, 95% confidence interval (CI): 0.87–1.51] and median progression-free survival (mPFS) [mPFS: 2.7 vs. 2.9 months, HR: 1.13, 95% CI: 0.89–1.43; objective response rates (ORR): 20.6% vs. 19.6%] were not improved compared with taxane. As for the safety of T-DM1, it had a lower incidence of grade ≥3 treatment-related adverse events (TRAEs). The most frequent grade ≥3 TRAEs were hematological diseases including anemia (26%) and thrombocytopenia (11%). There were eight (4%) deaths in the T-DM1 group and one death due to pulmonary alveolar hemorrhage (13) (**Table 2**). Similarly, for the subgroup analysis of Japanese patients screened in the GATSBY study, it was in line with the overall population and there was no improvement in survival benefits (mOS: 11.8 vs. 10.0 months, HR: 0.94, 95% CI: 0.52–1.72; mPFS: 2.3 vs. 2.9 months, HR: 1.33, 95% CI: 0.80–2.20; ORR: 22.5% vs. 33.3%) (26). In conclusion, T-DM1 did not show significant efficacy and safety in the treatment of HER2+ AGC patients. The failure of these results could be related to the high heterogeneity of HER2 expression and the difference in HER2 expression between metastatic and primary lesions.

**Table 2:** Data on the results of trials related to ADCs for gastric cancer.

Study	Trial group	Trial design	Primary endpoints	Secondary endpoints	Adverse reactions greater than or equal to grade 3
GATSBY (13) (NCT01641939)	Patients with previously treated locally advanced or metastatic HER2-positive gastric cancer	Trastuzumab emtansine; taxane	mOS: 7.9 vs. 8.6 months	mPFS: 2.7 vs. 2.9 months; mORR: 20.6% vs. 19.6%; mDOR: 4.3 vs. 3.7 months	Gr 3–4 TRAEs occurred in 60% and 70%, respectively.
DESTINY-Gastric01 (14) (NCT03329690)	Patients with HER2-positive advanced gastric or gastroesophageal junction adenocarcinoma	Trastuzumab deruxtecan; chemotherapy	mORR: 51% vs. 14%	mOS: 12.5 vs. 8.4 months; mPFS: 5.6 vs. 3.5 months	Gr 3 or higher TRAEs were decreased neutrophil count (51% vs. 24%), anemia (38% vs. 23%), and decreased white cell count (21% vs. 11%), respectively.
RC48 (15) (NCT03556345)	Patients with HER2-positive locally advanced or metastatic gastric or gastroesophageal junction	RC48-ADC	mORR: 24.8%	mPFS: 4.1 months; mOS: 7.9 months	Gr 3 or higher AEs occurred in 36.0%, and the RC48-related AEs were mainly decreased neutrophil count (3.2%).
TAK-264 (16) (NCT01577758)	Patients with GCC-expressing gastrointestinal malignancies	TAK-264	DLT: grade 4 neutropenia (4/19)	MTD: 1.8 mg/kg	Gr 3 or higher TRAEs were neutropenia (22%) and hypokalemia and febrile neutropenia (each with 7%).
IMMU-132-01 (17) (NCT01631552)	Patients with refractory metastatic epithelial cancers (CRC, SCLC, esophageal carcinoma, endometrial cancer, pancreatic ductal adenocarcinoma)	Sacituzumab govitecan	mORR: NA vs. 17.7% vs. NA vs. 22.2% vs. NA	mPFS: 3.9 vs. 3.7 vs. 3.4 vs. 3.2 vs. 2.0 months; mOS: 14.2 vs. 7.1 vs. 7.2 vs. 11.9 vs. 4.5 months	Gr 3 or higher TRAEs were neutropenia (42.4%) and febrile neutropenia (5.3%), respectively.

mOS, median overall survival; mPFS, median progression-free survival; mORR, median objective response rate; mDOR, median duration of response; Gr, grade; TRAEs, treatment-related adverse events; DLT, dose-limiting toxicities; MTD, maximum tolerated dose; NA, not available; CRC, colorectal cancer; SCLC, small-cell lung cancer.

#### Trastuzumab Deruxtecan

Trastuzumab deruxtecan (DS-8201a) has distinctive characteristics, which is an advantageous treatment option for patients with HER2-low expressing GC (27). The linker is enzymatically tetrapeptide-based and the upregulated lysosomal cathepsins can make the linker selectively cleaved. The payload, topoisomerase I inhibitor (DXd), is a superior derivative of exatecan. Its DAR is approximately 7–8 (28). Due to the remarkable results of the DESTINY-Breast01 trial (NCT03248492) (29), DS-8201a has been approved for the therapy of HER2+ breast cancer patients who have previously received at least two prior therapies in December 2019 (30).

There were various studies on DS-8201a as a treatment for HER2+ GC patients. A phase I trial (NCT02564900) was designed to evaluate the therapeutic efficacy and clinical safety of DS-8201a for AGC/GEJ patients. As for the safety profile, the most common any-grade AEs were concentrated on gastrointestinal reactions and hematological disorders including nausea (70%), decreased appetite (68%), and anemia (41%). The most frequently occurring grade ≥3 AEs were decreased neutrophil count (31%) and anemia (30%). The effectiveness of DS-8201a is shown by the following results: the ORR was 43.2% (95% CI: 28.3%–59.0%), the disease control rate (DCR) was 79.5% (95% CI: 64.7%–90.2%), the median time to response (mTTR) was 1.4 months (95% CI: 1.3–1.6), the median duration of response (mDOR) was 7.0 months (95% CI: 4.4–16.6), and the mPFS was 5.6 months (95% CI: 3.0–8.3) (28). From the above results, DS-8201a has demonstrated a relatively securable safety and preliminary antitumor ability. One hundred and eighty-seven HER2+ AGC/GEJ patients were enrolled in the DESTINY-Gastric01 study (NCT03329690) to receive DS-8201a (125 patients) or chemotherapy (62 patients). Compared with chemotherapy, DS-8201a improved the ORR (51% vs. 14%) and there were 10 patients with complete response (CR) and none in the chemotherapy group. DS-8201a significantly prolonged the survival time (mOS: 12.5 vs. 8.4 months, HR: 0.59, 95% CI: 0.39–0.88; mPFS: 5.6 vs. 3.5 months, HR: 0.47, 95% CI: 0.31–0.71). In terms of safety profile, the most frequent grade ≥3 AEs were abnormal hemograms such as decreased neutrophil count (51% vs. 24%), anemia (38% vs. 23%), and decreased white cell count (21% vs. 11%) (14) (**Table 2**). In addition, the DESTINY-Gastric02 (NCT04014075) and DESTINY-Gastric03 (NCT04379596) trials also showed good antitumor activity and safety (31). Based on the efficacy and safety of DS-8201a in the treatment of HER2+ AGC/GEJ patients, DS-8201a was approved by the FDA for the treatment of HER2+ GC/GEJ patients who were previously treated with trastuzumab in January 2021.

#### RC48

RC48 consists of the novel mAb hertuzumab, a cleavable dipeptide linker, and the microtubule inhibitor monomethyl auristatin E (MMAE) with a DAR of approximately 4 (32). MMAE plays a multifunctional role in inhibiting tubulin polymerization, resulting in efficient tumor regression. Hertuzumab has a superior affinity to other HER2-targeted mAbs which indicates a more efficient combination (33). It has been confirmed that RC48 can selectively deliver MMAE to the targeted tumor tissue in *in-vitro* and *in-vivo* trials. The targeted transport and release of RC48 can reduce systemic side effects and improve antitumor capacities in humans (34).

In a dose-escalation and dose-expansion phase I study (NCT02881190), RC48 was recognized as a prospectively tolerable and potential agent for HER2+ AGC therapy. The results indicated that the most tolerated dose (MTD) was uncertain, and the recommended phase 2 dose (RP2D) was 2.5 mg/kg. As for the toxicity analysis, the overall incidence of any-grade AEs was 94.7%. The dose-limiting toxicity (DLT) had dose-dependent properties and the incidence of DLT-associated AEs was 5.3%. The most generally occurring TRAEs were mild blood/lymphatic system/gastrointestinal disorders and the most common grade ≥3 TRAEs were neutropenia (19.3%), leukopenia (17.5%), and hypoesthesia (14.0%). It was worth mentioning that these GC patients (HER2: IHC2+/FISH− vs. IHC2+/FISH+ vs. IHC3+) had different responses (ORR: 35.7% vs. 20% vs. 13.6%), which implied that RC48 also improved the survival benefit of patients with HER2-low expressing GC (35). Furthermore, the results of a phase II trial (NCT03556345) showed that patients who received RC48 got prospective effectiveness (ORR: 24.8%, 95% CI: 17.5%–33.3%; mDOR: 4.7 months, 95% CI: 3.4–6.9 months; DCR: 42.4%, 95% CI: 33.6%–51.6%; mPFS: 4.1 months, 95% CI: 3.7–4.9 months; mOS: 7.9 months, 95% CI: 6.7–9.9 months). As reported, the incidence rates of any-grade and grade ≥3 AEs were 100% and 56.8%, respectively. There was no death associated with RC48 (15) (**Table 2**). In conclusion, RC48 demonstrated a magnificent antitumor activity and tolerable safety for HER2+ AGC patients who have received two lines of chemotherapy. Based on the impressive outcomes, RC48 successfully obtained conditional marketing approval by the China National Medical Products Administration (NMPA) for the treatment of locally advanced or metastatic GC/GEJ in June 2021.

### ADC Targeting GCC: TAK-264

GCC plays an important role in regulating intestinal fluid and ion secretion and inhibiting cell proliferation. GCC mRNA is overexpression in the peripheral blood and tumor tissues (36). It is the anatomically privileged localization that makes the high expression level of malignancy and a low expression level of normal tissue (37). TAK-264, a novel emerging ADC, consists of an IgG1 mAb, the payload MMAE, and a protease-cleavable linker (peptide maleimide-caproyl-valine-citrulline). TAK-264 has been investigated for its preliminary efficacy in killing GCC-overexpressing animal models (38).

The safety and clinical benefit of TAK-264 for advanced gastrointestinal cancer (GI) patients were investigated in a phase I study (NCT01577758). As reported, the MTD was 1.8 mg/kg and all patients had different grades of AEs. The most common TRAEs were nausea and decreased appetite (each with 41%), fatigue (32%), and diarrhea (27%). The most generally occurring grade ≥3 TRAEs included neutropenia (22%) and hypokalemia and febrile neutropenia (each with 7%). Of the 41 total enrolled patients, 1 patient achieved partial response, while 3 cases achieved stable disease. The mPFS of the overall population was 44 days (95% CI: 39–83). These outcomes indicated that TAK-264 had limited activity and a well-tolerated safety profile (16) (**Table 2**). Furthermore, a clinical trial (NCT02391038), designed to identify the efficacy of TAK-264 in Asian GI/GEJ patients, has reached similar conclusions (39). Even though the above findings demonstrated the finite clinical benefit of TAK-264, this agent was still full of prospects owing to its high expression frequency in GI cancer.

### ADCs Targeting Trop-2

Trop-2, also known as tumor-associated calcium signal transducer 2 (TACSTD2), has oncogenic properties which promote the process of tumor cell proliferation and metastasis. About 56% of GC patients who exhibited overexpression of Trop-2 were associated with a poor prognosis (40). Sacituzumab govitecan (IMMU-132) was the first Trop-2-targeted ADC approved by the FDA for the treatment of metastatic triple-negative breast cancer (mTNBC), which was comprised of the RS7, the payload SN-38, and a cleavable CL2A linker with a DAR of 7.6 (41–43).

A total of 495 patients with metastatic epithelial cancer (including gastric adenocarcinoma, mTNBC, and other 16 malignant tumors) received IMMU-132 in the IMMU-132-01 study (NCT01631552). The result of this phase I/II basket trial showed preliminary therapeutic activity (17, 44) (**Table 2**). SKB-264 is the other Trop-2-targeted ADC, which is optimized with proprietary cytotoxicity. It consists of a belotecan-derived payload and a novel stable conjugation chemistry with a DAR of 7.4. Preclinical research data indicated that SKB-264 had impressive anticancer activity in Trop-2-positive GC animal models, which also had good safety and tolerability (45). Further clinical trials are still needed to confirm the treatment effects on the Trop-2-overexpressing AGC patients.

### Other ADCs Under Development

CLDN18.2 participated in maintaining the tight junction between cells and affected the permeability of paracellular ions (46). Its characteristic of being a stomach-specific isoform made it a prospective target for the treatment of GC (47). LZ1904, a component of CLDN18.2, has shown significantly selective antitumor ability *in vitro*, which was worthy of further trials *in vivo*. However, the clinical trials of CLDN18.2 were in the initial stages (18).

The overexpression of HER3 in AGC was associated with unsatisfactory prognosis. EV20/NMS-P945, a novel ADC targeting HER3, was composed of the EV20; the thienoindole (TEI) NMS-P528, a derivative of doxorubicin; and a cleavable linker. The preclinical study found that EV20/NMS-P945 had good anticancer activity on GC cells and mouse xenograft tumor models, which indicated that this agent could be a potent tool against HER3-expressing AGC (19).

## Drug Resistance to ADCs

Drug resistance was the main hindrance to ADC treatment. The mechanism of resistance of ADCs was complex and diverse (48). Firstly, the downregulation or deletion of target antigens could be recognized by the mAb component of ADCs. Loganzo et al. proved that increasing the cycle exposure of ADCs could significantly reduce the level of antigen expression (49). In other aspects, van der Velden et al. illustrated that if drug exposure was reduced, the efficacy of ADC would be reduced due to the high expression of antigens (50). Secondly, ADCs released cytotoxic payloads through chemical or enzymatic cleavage functions in lysosomes. Pandiella et al. demonstrated that it was a disordered lysosomal proteolytic activity that resulted in drug resistance to T-DM1 (51). In addition, Hamblett et al. found that the reduction of lysosome transporters such as SLC46A3 inhibited the efficacy of numerous cytotoxic drugs (52). Thirdly, overexpression of drug efflux pumps was also associated with drug resistance (53). In addition, there were several potential mechanisms of resistance including internalization and transport pathways, apoptotic dysregulation, signaling pathways, etc. (54).

## Outlook and Summary

Gastric cancer is a highly heterogeneous tumor, which needs novel treatment methods to improve the prognosis of patients. HER2 heterogeneities of GC including organizational heterogeneity, spatial heterogeneity, and temporal heterogeneity were investigated (55). In recent years, immune checkpoint inhibitors and chimeric antigen receptor T cells (CAR-T) have shown positive efficacy in AGC. The regimen of pembrolizumab combined with trastuzumab and chemotherapy has received accelerated approval from the FDA based on its excellent effectiveness, which has become a new first-line anti-HER2 therapy for GC (56).

ADCs, emerging as innovative and potential anticancer drugs, have markedly improved the prognostic efficacy and medication safety in AGC patients (57). Multiple ADCs including DS-8201a and RC48 have achieved gratifying outcomes and were approved for the treatment of HER2-positive AGC. Meanwhile, preclinical studies and various clinical trials have indicated encouraging findings in different targets other than HER2 (58). Nevertheless, it was undeniable that several issues were supposed to be resolved. The limitations such as the AEs of ADCs were correlated with off-target payloads. It is worthy to optimize each ADC component (including antibody, conjugation site, linker, etc.) to improve its efficacy and safety (59).

In conclusion, ADCs are valid and well-tolerated anticancer drugs, whose development is a great breakthrough in tumor therapy and the linchpin of AGC treatment. In the future, optimizing each ADC component and better understanding potential modifications can make ADCs individualized and accurate. The research and development of ADCs will further improve the prognosis of AGC patients.

## Author Contributions

FK contributed the central idea and analyzed most of the data. NW wrote the initial draft of the paper. The remaining authors contributed to refining the ideas, carrying out additional analyses, and finalizing this paper.

## Funding

This work was supported by grants from the National Natural Science Foundation of China (No. 81403220), the National Key Research and Development (R&D) Plan (No. 2018YFC1707400), and the Tianjin Health and Family Planning-High Level Talent Selection and Training Project.

## Conflict of Interest

The authors declare that the research was conducted in the absence of any commercial or financial relationships that could be construed as a potential conflict of interest.

## Publisher’s Note

All claims expressed in this article are solely those of the authors and do not necessarily represent those of their affiliated organizations, or those of the publisher, the editors and the reviewers. Any product that may be evaluated in this article, or claim that may be made by its manufacturer, is not guaranteed or endorsed by the publisher.

## References

[1] Siegel RL Miller KD Fuchs HE Jemal A. Cancer Statistics, 2021. CA: Cancer J Clin (2021) 71:7–33. doi: 10.3322/caac.21654 33433946

[2] Joshi SS Badgwell BD. Current Treatment and Recent Progress in Gastric Cancer. CA: Cancer J Clin (2021) 71:264–79. doi: 10.3322/caac.21657 PMC9927927 33592120

[3] Fatima SW Khare SK. Benefits and Challenges of Antibody Drug Conjugates as Novel Form of Chemotherapy. J Controlled release (2022) 341:555–65. doi: 10.1016/j.jconrel.2021.12.013 34906604

[4] Williams M Spreafico A Vashisht K Hinrichs MJ. Patient Selection Strategies to Maximize Therapeutic Index of Antibody-Drug Conjugates: Prior Approaches and Future Directions. Mol Cancer Ther (2020) 19:1770–83. doi: 10.1158/1535-7163.MCT-19-0993 32546659

[5] Beck A Goetsch L Dumontet C Corvaïa N. Strategies and Challenges for the Next Generation of Antibody-Drug Conjugates. Nat Rev Drug Discov (2017) 16:315–37. doi: 10.1038/nrd.2016.268 28303026

[6] Ceci C Lacal PM Graziani G. Antibody-Drug Conjugates: Resurgent Anticancer Agents With Multi-Targeted Therapeutic Potential. Pharmacol Ther (2022) 236:108106. doi: 10.1016/j.pharmthera.2021.108106 34990642

[7] Drago JZ Modi S Chandarlapaty S. Unlocking the Potential of Antibody-Drug Conjugates for Cancer Therapy. Nat Rev Clin Oncol (2021) 18:327–44. doi: 10.1038/s41571-021-00470-8 PMC8287784 33558752

[8] Birrer MJ Moore KN Betella I Bates RC. Antibody-Drug Conjugate-Based Therapeutics: State of the Science. J Natl Cancer Inst (2019) 111:538–49. doi: 10.1093/jnci/djz035 30859213

[9] Weiner GJ. Building Better Monoclonal Antibody-Based Therapeutics. Nat Rev Cancer (2015) 15:361–70. doi: 10.1038/nrc3930 PMC4491443 25998715

[10] Wang X Mathieu M Brezski RJ. IgG Fc Engineering to Modulate Antibody Effector Functions. Protein Cell (2018) 9:63–73. doi: 10.1007/s13238-017-0473-8 28986820 PMC5777978

[11] Tarantino P Carmagnani Pestana R Corti C Modi S Bardia A Tolaney SM. Antibody-Drug Conjugates: Smart Chemotherapy Delivery Across Tumor Histologies. CA: Cancer J Clin (2021) 72:165–82. doi: 10.3322/caac.21705 34767258

[12] Koganemaru S Shitara K. Antibody-Drug Conjugates to Treat Gastric Cancer. Expert Opin Biol Ther (2021) 21:923–30. doi: 10.1080/14712598.2020.1802423 32713216

[13] Thuss-Patience PC Shah MA Ohtsu A Van Cutsem E Ajani JA Castro H. Trastuzumab Emtansine Versus Taxane Use for Previously Treated HER2-Positive Locally Advanced or Metastatic Gastric or Gastro-Oesophageal Junction Adenocarcinoma (GATSBY): An International Randomised, Open-Label, Adaptive, Phase 2/3 Study. Lancet Oncol (2017) 18:640–53. doi: 10.1016/S1470-2045(17)30111-0 28343975

[14] Shitara K Bang YJ Iwasa S Sugimoto N Ryu MH Sakai D. Trastuzumab Deruxtecan in Previously Treated HER2-Positive Gastric Cancer. N Engl J Med (2020) 382:2419–30. doi: 10.1056/NEJMoa2004413 32469182

[15] Peng Z Liu T Wei J Wang A He Y Yang L. Efficacy and Safety of a Novel Anti-HER2 Therapeutic Antibody RC48 in Patients With HER2-Overexpressing, Locally Advanced or Metastatic Gastric or Gastroesophageal Junction Cancer: A Single-Arm Phase II Study. Cancer Commun (London England) (2021) 41:1173–82. doi: 10.1002/cac2.12214 PMC8626607 34665942

[16] Almhanna K Kalebic T Cruz C Faris JE Ryan DP Jung J. Phase I Study of the Investigational Anti-Guanylyl Cyclase Antibody-Drug Conjugate TAK-264 (MLN0264) in Adult Patients With Advanced Gastrointestinal Malignancies. Clin Cancer Res (2016) 22:5049–57. doi: 10.1158/1078-0432.CCR-15-2474 27178743

[17] Bardia A Messersmith WA Kio EA Berlin JD Vahdat L Masters GA. Sacituzumab Govitecan, a Trop-2-Directed Antibody-Drug Conjugate, for Patients With Epithelial Cancer: Final Safety and Efficacy Results From the Phase I/II IMMU-132-01 Basket Trial. Ann Oncol (2021) 32:746–56. doi: 10.1016/j.annonc.2021.03.005 33741442

[18] Zhang J Dong R Shen L. Evaluation and Reflection on Claudin 18.2 Targeting Therapy in Advanced Gastric Cancer. Chin J Cancer Res = Chung-kuo yen cheng yen chiu (2020) 32:263–70. doi: 10.21147/j.issn.1000-9604.2020.02.13 PMC7219097 32410803

[19] Capone E Lattanzio R Gasparri F Orsini P Rossi C Iacobelli V. EV20/NMS-P945, a Novel Thienoindole Based Antibody-Drug Conjugate Targeting HER-3 for Solid Tumors. Pharmaceutics (2021) 13:483. doi: 10.3390/pharmaceutics13040483 33918158 PMC8066800

[20] Roviello G Aprile G D'Angelo A Iannone LF Roviello F Polom K. Human Epidermal Growth Factor Receptor 2 (HER2) in Advanced Gastric Cancer: Where do We Stand? Gastric Cancer (2021) 24:765–79. doi: 10.1007/s10120-021-01182-9 33742317

[21] Zhang J Fan J Zeng X Nie M Chen W Wang Y. Targeting the Autophagy Promoted Antitumor Effect of T-DM1 on HER2-Positive Gastric Cancer. Cell Death Dis (2021) 12:288. doi: 10.1038/s41419-020-03349-1 33731670 PMC7969610

[22] Yamashita-Kashima Y Shu S Harada N Fujimoto-Ouchi K. Enhanced Antitumor Activity of Trastuzumab Emtansine (T-DM1) in Combination With Pertuzumab in a HER2-Positive Gastric Cancer Model. Oncol Rep (2013) 30:1087–93. doi: 10.3892/or.2013.2547 23783223

[23] Xu Z Guo D Jiang Z Tong R Jiang P Bai L. Novel HER2-Targeting Antibody-Drug Conjugates of Trastuzumab Beyond T-DM1 in Breast Cancer: Trastuzumab Deruxtecan(DS-8201a) and (Vic-)Trastuzumab Duocarmazine (Syd985). Eur J Med Chem (2019) 183:111682. doi: 10.1016/j.ejmech.2019.111682 31563805

[24] Krop IE Kim S-B Martin AG LoRusso PM Ferrero J-M Badovinac-Crnjevic T. Trastuzumab Emtansine Versus Treatment of Physician's Choice in Patients With Previously Treated HER2-Positive Metastatic Breast Cancer (TH3RESA): Final Overall Survival Results From a Randomised Open-Label Phase 3 Trial. Lancet Oncol (2017) 18:743–54. doi: 10.1016/S1470-2045(17)30313-3 28526538

[25] Diéras V Miles D Verma S Pegram M Welslau M Baselga J. Trastuzumab Emtansine Versus Capecitabine Plus Lapatinib in Patients With Previously Treated HER2-Positive Advanced Breast Cancer (EMILIA): A Descriptive Analysis of Final Overall Survival Results From a Randomised, Open-Label, Phase 3 Trial. Lancet Oncol (2017) 18:732–42. doi: 10.1016/S1470-2045(17)30312-1 PMC5531181 28526536

[26] Shitara K Honma Y Omuro Y Yamaguchi K Chin K Muro K. Efficacy of Trastuzumab Emtansine in Japanese Patients With Previously Treated HER2-Positive Locally Advanced or Metastatic Gastric or Gastroesophageal Junction Adenocarcinoma: A Subgroup Analysis of the GATSBY Study. Asia-Pacific J Clin Oncol (2020) 16:5–13. doi: 10.1111/ajco.13243 31721447

[27] Takegawa N Nonagase Y Yonesaka K Sakai K Maenishi O Ogitani Y. DS-8201a, a New HER2-Targeting Antibody-Drug Conjugate Incorporating a Novel DNA Topoisomerase I Inhibitor, Overcomes HER2-Positive Gastric Cancer T-DM1 Resistance. Int J Cancer (2017) 141:1682–9. doi: 10.1002/ijc.30870 28677116

[28] Shitara K Iwata H Takahashi S Tamura K Park H Modi S. Trastuzumab Deruxtecan (DS-8201a) in Patients With Advanced HER2-Positive Gastric Cancer: A Dose-Expansion, Phase 1 Study. Lancet Oncol (2019) 20:827–36. doi: 10.1016/S1470-2045(19)30088-9 31047804

[29] Modi S Saura C Yamashita T Park YH Kim SB Tamura K. Trastuzumab Deruxtecan in Previously Treated HER2-Positive Breast Cancer. N Engl J Med (2020) 382:610–21. doi: 10.1056/NEJMoa1914510 PMC7458671 31825192

[30] Keam SJ. Trastuzumab Deruxtecan: First Approval. Drugs (2020) 80:501–8. doi: 10.1007/s40265-020-01281-4 32144719

[31] Aoki M Iwasa S Boku N. Trastuzumab Deruxtecan for the Treatment of HER2-Positive Advanced Gastric Cancer: A Clinical Perspective. Gastric Cancer (2021) 24:567–76. doi: 10.1007/s10120-021-01164-x 33646464

[32] Li L Xu MZ Wang L Jiang J Dong LH Chen F. Conjugating MMAE to a Novel Anti-HER2 Antibody for Selective Targeted Delivery. Eur Rev Med Pharmacol Sci (2020) 24:12929–37. doi: 10.26355/eurrev_202012_24196 33378043

[33] Rinnerthaler G Gampenrieder SP Greil R. HER2 Directed Antibody-Drug-Conjugates Beyond T-DM1 in Breast Cancer. Int J Mol Sci (2019) 20:1115. doi: 10.3390/ijms20051115 PMC6429068 30841523

[34] Chen Z Yuan J Xu Y Zhang C Li Z Gong J. From AVATAR Mice to Patients: RC48-ADC Exerted Promising Efficacy in Advanced Gastric Cancer With HER2 Expression. Front Pharmacol (2021) 12:757994. doi: 10.3389/fphar.2021.757994 35069192 PMC8769204

[35] Xu Y Wang Y Gong J Zhang X Peng Z Sheng X. Phase I Study of the Recombinant Humanized Anti-HER2 Monoclonal Antibody-MMAE Conjugate RC48-ADC in Patients With HER2-Positive Advanced Solid Tumors. Gastric Cancer (2021) 24:913–25. doi: 10.1007/s10120-021-01168-7 PMC8205919 33945049

[36] Bose A Banerjee S Visweswariah SS. Mutational Landscape of Receptor Guanylyl Cyclase C: Functional Analysis and Disease-Related Mutations. IUBMB Life (2020) 72:1145–59. doi: 10.1002/iub.2283 PMC7611479 32293781

[37] Almhanna K Prithviraj GK Veiby P Kalebic T. Antibody-Drug Conjugate Directed Against the Guanylyl Cyclase Antigen for the Treatment of Gastrointestinal Malignancies. Pharmacol Ther (2017) 170:8–13. doi: 10.1016/j.pharmthera.2016.10.007 27765652

[38] Gallery M Zhang J Bradley DP Brauer P Cvet D Estevam J. A Monomethyl Auristatin E-Conjugated Antibody to Guanylyl Cyclase C Is Cytotoxic to Target-Expressing Cells *In Vitro* and *In Vivo* . PloS One (2018) 13:e0191046. doi: 10.1371/journal.pone.0191046 29370189 PMC5784926

[39] Bang YJ Takano T Lin CC Fasanmade A Yang H Danaee H. TAK-264 (MLN0264) in Previously Treated Asian Patients With Advanced Gastrointestinal Carcinoma Expressing Guanylyl Cyclase C: Results From an Open-Label, Non-Randomized Phase 1 Study. Cancer Res Treat (2018) 50:398–404. doi: 10.4143/crt.2017.074 28494535 PMC5912138

[40] Cardillo TM Govindan SV Sharkey RM Trisal P Arrojo R Liu D. Sacituzumab Govitecan (IMMU-132), an Anti-Trop-2/SN-38 Antibody-Drug Conjugate: Characterization and Efficacy in Pancreatic, Gastric, and Other Cancers. Bioconjugate Chem (2015) 26:919–31. doi: 10.1021/acs.bioconjchem.5b00223 25915780

[41] Bardia A Mayer IA Diamond JR Moroose RL Isakoff SJ Starodub AN. Efficacy and Safety of Anti-Trop-2 Antibody Drug Conjugate Sacituzumab Govitecan (IMMU-132) in Heavily Pretreated Patients With Metastatic Triple-Negative Breast Cancer. J Clin Oncol (2017) 35:2141–8. doi: 10.1200/JCO.2016.70.8297 PMC5559902 28291390

[42] Ocean AJ Starodub AN Bardia A Vahdat LT Isakoff SJ Guarino M. Sacituzumab Govitecan (IMMU-132), an Anti-Trop-2-SN-38 Antibody-Drug Conjugate for the Treatment of Diverse Epithelial Cancers: Safety and Pharmacokinetics. Cancer (2017) 123:3843–54. doi: 10.1002/cncr.30789 28558150

[43] Syed YY. Sacituzumab Govitecan: First Approval. Drugs (2020) 80:1019–25. doi: 10.1007/s40265-020-01337-5 PMC7288263 32529410

[44] Sharkey RM McBride WJ Cardillo TM Govindan SV Wang Y Rossi EA. Enhanced Delivery of SN-38 to Human Tumor Xenografts With an Anti-Trop-2-SN-38 Antibody Conjugate (Sacituzumab Govitecan). Clin Cancer Res (2015) 21:5131–8. doi: 10.1158/1078-0432.CCR-15-0670 26106073

[45] Zaman S Jadid H Denson AC Gray JE. Targeting Trop-2 in Solid Tumors: Future Prospects. OncoTargets Ther (2019) 12:1781–90. doi: 10.2147/OTT.S162447 PMC6402435 30881031

[46] Pellino A Brignola S Riello E Niero M Murgioni S Guido M. Association of CLDN18 Protein Expression With Clinicopathological Features and Prognosis in Advanced Gastric and Gastroesophageal Junction Adenocarcinomas. J Pers Med (2021) 11:1095. doi: 10.3390/jpm11111095 34834447 PMC8624955

[47] Rohde C Yamaguchi R Mukhina S Sahin U Itoh K Türeci Ö. Comparison of Claudin 18.2 Expression in Primary Tumors and Lymph Node Metastases in Japanese Patients With Gastric Adenocarcinoma. Japanese J Clin Oncol (2019) 49:870–6. doi: 10.1093/jjco/hyz068 PMC6792344 31087075

[48] Collins DM Bossenmaier B Kollmorgen G Niederfellner G. Acquired Resistance to Antibody-Drug Conjugates. Cancers (2019) 11:394. doi: 10.3390/cancers11030394 PMC6468698 30897808

[49] Loganzo F Tan X Sung M Jin G Myers JS Melamud E. Tumor Cells Chronically Treated With a Trastuzumab-Maytansinoid Antibody-Drug Conjugate Develop Varied Resistance Mechanisms But Respond to Alternate Treatments. Mol Cancer Ther (2015) 14:952–63. doi: 10.1158/1535-7163.MCT-14-0862 25646013

[50] van der Velden VH Boeckx N Jedema I te Marvelde JG Hoogeveen PG Boogaerts M. High CD33-Antigen Loads in Peripheral Blood Limit the Efficacy of Gemtuzumab Ozogamicin (Mylotarg) Treatment in Acute Myeloid Leukemia Patients. Leukemia (2004) 18:983–8. doi: 10.1038/sj.leu.2403350 15029214

[51] Rios-Luci C Garcia-Alonso S Diaz-Rodriguez E Nadal-Serrano M Arribas J Ocana A. Resistance to the Antibody-Drug Conjugate T-DM1 Is Based in a Reduction in Lysosomal Proteolytic Activity. Cancer Res (2017) 77:4639–51. doi: 10.1158/0008-5472.CAN-16-3127 28687619

[52] Hamblett KJ Jacob AP Gurgel JL Tometsko ME Rock BM Patel SK. SLC46A3 Is Required to Transport Catabolites of Noncleavable Antibody Maytansine Conjugates From the Lysosome to the Cytoplasm. Cancer Res (2015) 75:5329–40. doi: 10.1158/0008-5472.CAN-15-1610 26631267

[53] Kovtun YV Audette CA Mayo MF Jones GE Doherty H Maloney EK. Antibody-Maytansinoid Conjugates Designed to Bypass Multidrug Resistance. Cancer Res (2010) 70:2528–37. doi: 10.1158/0008-5472.CAN-09-3546 20197459

[54] García-Alonso S Ocaña A Pandiella A. Resistance to Antibody-Drug Conjugates. Cancer Res (2018) 78:2159–65. doi: 10.1158/0008-5472.CAN-17-3671 29653942

[55] Zhang H Wang Y Wang Y Wu D Lin E Xia Q. Intratumoral and Intertumoral Heterogeneity of HER2 Immunohistochemical Expression in Gastric Cancer. Pathol Res Pract (2020) 216:153229. doi: 10.1016/j.prp.2020.153229 33010699

[56] Janjigian YY Kawazoe A Yañez P Li N Lonardi S Kolesnik O. The KEYNOTE-811 Trial of Dual PD-1 and HER2 Blockade in HER2-Positive Gastric Cancer. Nature (2021) 600:727–30. doi: 10.1038/s41586-021-04161-3 PMC8959470 34912120

[57] Khongorzul P Ling CJ Khan FU Ihsan AU Zhang J. Antibody-Drug Conjugates: A Comprehensive Review. Mol Cancer Res (2020) 18:3–19. doi: 10.1158/1541-7786.MCR-19-0582 31659006

[58] Thomas A Teicher BA Hassan R. Antibody-Drug Conjugates for Cancer Therapy. Lancet Oncol (2016) 17:e254–62. doi: 10.1016/S1470-2045(16)30030-4 PMC6601617 27299281

[59] Abdollahpour-Alitappeh M Lotfinia M Gharibi T Mardaneh J Farhadihosseinabadi B Larki P. Antibody-Drug Conjugates (ADCs) for Cancer Therapy: Strategies, Challenges, and Successes. J Cell Physiol (2019) 234:5628–42. doi: 10.1002/jcp.27419 30478951

